# Treatment of depression by traditional faith healers in Nepal: A qualitative study

**DOI:** 10.1016/j.ssmmh.2025.100425

**Published:** 2025-06

**Authors:** Nagendra P. Luitel, Bishnu Lamichhane, Prakriti Koirala, Poonam Sainju, Amshu Ghimire, Kamal Gautam, Mark JD. Jordans, Brandon A. Kohrt

**Affiliations:** a Research Department, Transcultural Psychosocial Organization (TPO) Nepal, Baluwatar, Kathmandu, Nepal; b Center for Global Mental Health Equity, Department of Psychiatry and Behavioural Health, The George Washington University, Washington, D.C., USA; c Centre for Global Mental Health, Health Service and Population Research Department, Institute of Psychiatry, Psychology and Neuroscience, King's College London, London, UK

**Keywords:** Traditional faith healers, Perceived causes, Treatment process, Depression, Nepal

## Abstract

This study explored the assessment and treatment practices of traditional faith healers for patients with depression, as well as their beliefs about the causes of depression, in Nepal. In-depth interviews were conducted using a narrative approach to allow participants to share their experiences and perspectives through storytelling. A vignette depicting a woman with depression symptoms was used to investigate traditional faith healers' beliefs about the character's health issues. The interview guide included questions about the assessment and treatment procedures of traditional faith healers for the character in the vignette. The interviews were conducted between January and July 2023 with 12 traditional faith healers. Traditional faith healers hold diverse beliefs about the causes of depression. Some attribute it to supernatural forces or curses, such as the spirit of a husband or ancestor, while others link it to mental stress, personal and social factors, and lack of support. They employ various methods to assess and treat depression, including examining the patient's face, using rice grains, invoking spirits, creating statues from clay or wheat flour, employing blowing techniques, listening to their suffering, performing death rituals to remove ancestral spirits, shouting, and referring them to hospitals. Collaborating with traditional healers, who act as gateways to mental health care, could improve early detection and treatment of depression. Some traditional healing practices may be helpful for depression, so involving them in identifying and supporting individuals with depression could be beneficial. Future research should evaluate the effectiveness of traditional healing treatments and explore patients' views on these treatments.

## Highlights

• People with mental health issues in Nepal often seek help from traditional healers, however, little is known about how they treat these issues.
• This study employed a narrative approach to explore the assessment and treatment methods of traditional healers for depression.
• Healers have various beliefs about the causes of depression, such as supernatural forces, mental stress, and personal and social factors.
• They use several methods to assess and treat depression, including facial examination, using rice grain, statue creation and blowing techniques.
• Collaborating with traditional healers, who act as gateways to mental health care, could enhance early detection and treatment of depression.

## 1 Introduction

Despite the availability of evidence-based treatments for mental health conditions, a large majority (86.3 %) of individuals in lower-middle-income countries do not receive biomedical treatment for anxiety, mood or substance disorders (Evans-Lacko et al., 2018). Research shows that people with depression often seek help from traditional faith healers, friends or family members instead of biomedical professionals like doctors, psychiatrists, or psychologists (Rickwood and Braithwaite, 1994; Rickwood et al., 2007). The use of complementary approaches to treat mental health is a common global phenomenon (Budžak and Branković, 2022). Faith healers have traditionally played a crucial role in mental health care in many regions, often being the first point of contact for individuals seeking assistance with mental health issues (Nortje et al., 2016). In developing countries, many people choose traditional healers for mental health concerns due to cultural beliefs associating mental health problems with supernatural causes such as witchcraft or sins from past lives. This preference is reinforced by the trust and familiarity individuals have with traditional healers who share their community, culture, and beliefs (Anjorin and Wada, 2022; Kganya, 2023; Green and Colucci, 2020). Additionally, traditional healers offer care for rural populations without access to formal healthcare services and provide a more affordable option for mental health care in urban areas where formal services are costly (WHO, 2021). Traditional healers, thus, serve multiple roles in mental health including potentially delivering effective treatment and identifying people with mental health conditions who could benefit from referral to biomedical mental health services. Given the potential benefit of some forms of traditional healing, some have argued that availability of traditional faith healers should be a consideration when estimating the mental health treatment gap (Pham et al., 2021c).

Although there are claims that traditional healing practices in Nepal are on the decline (Baniya, 2014), more than 50% of the rural population still rely on traditional or complementary medicines (Shankar and Paudel, 2006). The treatment of mental health issues by traditional healers therefore remains relatively common in Nepal, possibly due to various reasons such as a strong belief in spiritual imbalances as the cause of mental illness (Atreya et al., 2023), affordability compared to medical treatment (Pandey, 2024; Thapa et al., 2018), cultural concept of distress (Kohrt and Hruschka, 2010), faith in traditional healers (Thapa et al., 2019), accessibility and reduced stigmatization (Atreya et al., 2023), and familiarity and trust (Thapa et al., 2019).

Despite extensive literature detailing the preferences and roles of traditional healers in various cultural contexts, there remains a significant gap in understanding the specific methods and procedures they employ for assessing and treating mental health conditions (Nortje et al., 2016; Pham et al., 2021a). The diversity of traditional healing practices complicates the evaluation of mental health services provided by traditional healers, highlighting the need for more systematic data on this relationship. In Nepal, traditional healers consider their treatments effective for mild to moderate psychological distress and advocate for collaboration with biomedical providers through a two-way referral system (Pham et al., 2021b). Conversely, studies have documented negative consequences of seeking treatment for depression from traditional healers, such as harmful practices like physical restraint or isolation, delays in receiving evidence-based medical treatment, and the potential for worsening symptoms due to inappropriate or ineffective interventions (Anjorin and Hassan Wada, 2022).

This study aims to address these research gaps by exploring the assessment and treatment methods employed by traditional healers for patients with depression, as well as their beliefs about the underlying causes of depression. The results of this study will have practical implications for identifying effective traditional healing practices for treating depression and integrating them into formal mental health care systems. If traditional healers can detect illnesses in the early stages, it could facilitate early management of cases and ensure appropriate referrals. Understanding traditional healers' treatment practices can help policymakers develop strategies to support these practitioners and exploring engagement of traditional healers in detecting, managing, and referring patients with mental health problems to the existing healthcare system. By gaining a deeper understanding of traditional healing practices, particularly how they detect and manage depression, we can develop a more holistic and culturally sensitive mental health care approach to minimize the treatment gap for mental health conditions in Nepal and other low resource settings.

## 2 Methods

### 2.1 Setting

Nepal is a low-income country located in South Asia with a total population of approximately 29.1 million and an annual growth rate of 0.92%. The country is diverse in terms of caste/ethnicity and languages, with 142 caste ethnicities and 124 languages. The study was conducted in Jhapa, Chitwan, and Kailali districts, situated in the eastern, central, and western parts of Nepal, respectively. The total populations of these districts are 994,090, 722,168, and 911,155, respectively. Each district is characterized by diversity in caste/ethnicity, language, religion, and geography. Jhapa has a higher population of Brahman/Chhetri, Rai, Limbu, and indigenous communities such as Rajbanshi, Santhal, and Dhimal, while Chitwan has a significant Tamang, Darai and Magar population, and Kailali is predominantly inhabited by Tharu people. In Nepal, each caste/ethnic group has its own traditional beliefs and practices in the treatment of illnesses and has traditional providers such as Lamas, Bijuwa, Guruwas, Kul-Dhamis, and Matas. Primary health care services in Nepal are provided free of charge to patients through basic health centers, health posts, primary health care centers, and municipal hospitals.

### 2.2 Research design

The study employed a qualitative exploratory design, conducting individual in-depth interviews (IDI) using a narrative approach. Narrative interviewing is a qualitative research method that involves prompting interviewees to share their experiences and perspectives through storytelling. It is conducted as a conversation where participants freely discuss their experiences, with the researcher guiding the discussion to explore the research topic (Jovchelovitch Sandra & Bauer MW, 2000).

### 2.3 Participants and recruitment

The study involved 12 traditional faith healers selected through purposive sampling based on predefined criteria. Participants were chosen based on community recommendations, considering their popularity, diverse castes/ethnicities (Mata, Jyotish, Chaudhari Guruwa, Rajbanshi Guruwa, Lama Dhami, Limbu Dhami, and Kul-Dhami), and experience in treating symptoms described in the vignette. The traditional healers interviewed were from Chitwan (N = 3), Jhapa (N = 7), and Kailali (N = 2). Out of the 12 traditional healers, two (one from Jhapa and one from Chitwan) had prior training in detecting of mental health problems using the Community Informant Detection Tool (CIDT).

### 2.4 Interview guides

A vignette featuring a woman with depression was used to explore traditional healers' beliefs about the causes of her problems. The interview guide included questions about the assessment and treatment process of traditional healers for the character in the vignette. The vignette was adapted from the Community Informant Detection Tool (CIDT) developed (Subba et al., 2017) and validated (Jordans et al., 2015) in Nepal. Previous studies have demonstrated that narrative interviews are an effective method for gathering participants' perspectives on sensitive topics such as mental health issues (Lapatin et al., 2012). Traditional healers were asked about their assessment and treatment approaches for patients described in the vignette. The vignette and interview questions are included in the box below.

Box-1 **interview guides**

**Interview guides**.

*Laxmi is 36 years old married woman. She has two daughters and a son. For a while, she has been looking very unhappy and sad. Now-a-days she does not want to do usual works and she does not feel like working. Because she cannot fall sleep at night, she feels very weak and she gets tired easily. Recently she does not like to do anything. She thinks that she can do nothing in her life. She feels that her future is not bright, so she sometimes does not want to live anymore or thinks that her life is worthless. She had these thoughts since last year after the death of her husband. For the past weeks she has been rarely working in field and staying alone at home for a whole day*.

*Laxmi, a 36-year-old married woman with two daughters and a son, has been feeling increasingly unhappy and sad. She has lost interest in her usual activities and feels fatigued due to difficulty falling asleep at night. Laxmi has been experiencing negative thoughts about her future and sometimes questions the value of her life, especially since the death of her husband last year. Recently, she has been avoiding work in the fields and spending most of her time alone at home*.

1. In your opinion, is the character of this story, Laxmi, having a problem or hard time? How problematic/hard is her situation? In your opinion, what exactly is problem for Laxmi?
2. What kind of problem or illness is Laxmi facing or having?
3. In your community/village what kind of name (s) is given to this kind of illness? What else? How do we identify this illness?
4. Where (in her body) is she experiencing her problem (Inquire about *Man* and body relation)? Can you explain?
5. Why is Laxmi having this problem (Reasons)? (Inquire about Thoughts/Emotion, Society, *Laago/*Ghost etc)
6. In your opinion, can Laxmi's illness be healed or not? Why?
7. What type of people have higher probability of having these problems? Why?
8. Have you meet anyone in the community who had similar problems like Laxmi and recovered from it? Can they help Laxmi in her treatment? If yes, how?

*Laxmi is also not opening up and sharing her problems with others. She always complains about having problems but she does not want to discuss it openly. For the past few days, she is unable to do her usual work (household chores, working on the field). Even though she is having problems, she is not going for treatment*.

1. In your opinion, why is Laxmi not sharing about her problems with others?
2. If she had shared about her problems to others, what would they say about it?
3. Though Laxmi is having problem/hard times, why is she not going for treatment?
4. What kind of treatment or help does she need? Where and whom should she visit? Why?
5. Where would you suggest Laxmi to go for treatment and why? (Traditional healer, *Bhaakal,* Doctor)
6. What would influence or facilitate Laxmi's decision to go for treatment?
7. Do you think Laxmi's illness can be treated?
8. If Laxmi does not receive timely treatment, then what will be her condition?
9. Why is Laxmi unwilling to go to health post or district hospital for treatment? (Inquire about all reasons)
10. How could you convince Laxmi to get treatment? Who will she listen to in this community? In your opinion, what will be the responsibility of family in her treatment?

Alt-text: Box-1

### 2.5 Interview process

The interviews were conducted between January and July 2023 by two experienced qualitative researchers with Master's degrees. Written informed consent was obtained from each participant, outlining the study's aims, recruitment criteria, process, potential risks and benefits, confidentiality, and data usage. Only participants who provided consent were included in the study, and participation was voluntary. Participants were informed of their right to withdraw from the study at any time without explanation. All selected participants took part in the interviews.

Traditional healers, such as Dhami-Jhankri (shamans believed to communicate with spirits and deities), Jyotish (astrologers who predict events based on stars and recommend rituals like “grah-shanti," for household peace), and Mata (female healers, often referred to a woman who had become possessed by a spirit that in turn gave her healing powers), were initially screened community referrals. Interviews took place at spiritual sites (Nepali: *asan*) where healers treat patients often connected to their residences. The interviews were recorded with the participants' consent, lasting between 1 and 1.5 h based on the healers' willingness to share their experiences. Researchers observed the registration process and the treatment order of patients by traditional healers. It is important to mentioned that interruptions occurred during the interviews as clients arrived for services. If a healer was possessed by their ancestral deity (Nepali: *kul-devta*), the formal interview was paused during this time.

### 2.6 Data analysis and interpretation

All interviews were audio recorded and transcribed in Nepali by the interviewers. These transcriptions were then translated into English by professional translators. The data was analyzed using a combination of content and thematic analysis. Two researchers independently read the transcripts to familiarize themselves with the data and develop a thematic framework. Subsequently, 10% of the interviews were coded separately in NVivo 20 using the developed coding framework. The inter-coder agreement between the two researchers was measured, and a significant level of agreement was achieved. The thematic coding framework was finalized after discussion with other members of the research team. All interviews were indexed and charted based on the finalized framework, and the data from the charts was interpreted using the content analysis method.

## 3 Results

Of the total 12 traditional healers, the 9 were male, belonging to the Brahman/Chhetri (N = 6) and Janajati caste/ethnicity (N = 4), and all were married. The age of the participants ranged from 32 to 70 years (Median = 49.2). Five traditional healers had a secondary or higher level of education, while four were only literate.

Table 1 summarizes the perceptions of traditional healers regarding the causes, assessment (diagnostic process), and treatment. The results show that traditional healers have varied perspectives on the illness, its causes, and the assessment and management processes based on their beliefs about the causes of illness and the types of gods they believe in.

**Table 1:** Perceived causes, assessment, and treatment of depression.

Code, Gender	Type of traditional healers	Cause	Assessment (diagnostic process) and treatment process
KPPS_12, man	Traditional healer of Tamang community *(lama)*	- Afflicted by ghosts/spirits - Stress and tension	- Ritual blowing (*jharphuk*) - Creating peace and happiness in mind
KPBL_12, woman	Female traditional healer (*mata*)	- Supernatural power (*mashaan, pichaash, bira*)	- Communicating with the evil spirit causing the issues - Fulfilling the desires of evil spirit
KPBL_03, man	General traditional healer (*dhami*)	- Ghost or spirit of a dead person *(bayu)*	- Using grains and prayer - Calling the ghost/evil spirit in ill person's body and fulfilling their demand
KPBL_43 man	General traditional healer (*dhami*)	- Chudelni (the woman who died not being able to give birth to a child)	- Rituals blowing (phukne) repeatedly using a pair of pigeon
KPBL-13 man	Astrologer (j*yotish*)	- Mental stress/mental health problems - Affected by planets - Evil eyes (*nazar lageko*)	- Analysing planet's position against her date of birth - Referring her to doctor/hospital/PHC - Using smoke made from mustered, garlic and onion
KPBL_21, man	Clan traditional healer (*kul dhami*)	- Clan God (*devata*) - Over thinking or worry - Depression or Hysteria	- Referring to a doctor/psychiatrist - Advising to engage in fun activities and engage in day-to-day work
KPBL-42, man	General traditional healer (*dhami*)	- Curse of ancestors or husband *(pitri-dosh)* - Goddess of the forest (*kali, jangali)*	- Performing ritual to help achieve salvation of their ancestors - Using grains and various materials to remove bad energy from forest goddess (*kali, jangali*)
KPBL_40, man	Traditional healer of Limbu caste (*bijuwa*)	- Husband's unrest spirit -Birth chart (*kundali*)	- Performing Hindu ritual to manage salvation - Using some plants and food
KPBL-22, woman	Female traditional healer (*mata*)	- Personal factors *e.g.* low-self stream - Lack of family and social support	- Counseling to build self-esteem and self-confidence - Enhancing family and community support
KPBL-05, man	Traditional healer of Tharu community (*guruwa*)	- Menstrual disorder - Personal reasons (heart-mind problem)	- Creating a clay statue of a person with a menstrual disorder and worship - Nothing to do for heart-mind problems (*manko samasya)*
KPBL_44, woman	Female traditional healer (*mata*)	- Mental stress or tension	- Referring to doctor/hospital - Offering traditional healing practices
KPBL_41, man	Clan traditional healer (*kul dhami*)	- Mental illness due to grief from husband's death - Over thinking due to hardship	- Referring to mental hospital/psychologists or psychiatrist *(manasik doctors)*

### 3.1 Perceived causes of depression

Traditional healers identified several factors that could contribute to the depression experienced by the character in the vignette. The most commonly reported causes included being possessed by supernatural or spiritual powers, the influence of a husband's or ancestral spirit (pitri dosh), traumatic experiences, personal and social factors, mental stress or tension, and excessive rumination.

#### 3.1.1 Supernatural power (mashaan, pichaash, chudelni, bayu, jangali)

Some traditional healers believed that the person described in the vignette was possessed by ghosts (*bhut*) and other supernatural entities *(mashaan, pichaash, bayu*). These spirits are believed to be the souls of individuals who died unnaturally or at a young age, and did not find peace after death.

A female traditional healer (Mata) from Chitwan reported that the individual was experiencing various issues, such as trouble sleeping, due to the presence of a restless spirit of a person (*mashaan, pichaash*) who died at a young age and has not found peace in the afterlife.

*If she frequently feels tired and has trouble sleeping at night, it is because of restless spirits (*mashaan, pichaash)*. Some people die at a young age and are unable to find peace after death, becoming trapped and causing harm to others. They are known as* mashaan, pichaash, *or* bira*. Those who are possessed often experience fatigue, insomnia, and lethargy. If individuals seek medical help, doctors may diagnose them with a mental health issue or weakness.***[Mata]**

The same traditional healer mentioned that the *pichaash* can create a situation where the possessed person experiences severe distress, sometimes leading to suicidal thoughts or attempts.

*When a* pichaash *possesses a person, it can cause severe distress, leading to thoughts of suicide, mental instability, or family discord. The* pichaash *can manifest in different forms such as people, snakes, or gods, and may try to engage in physical intimacy with the possessed individual, making it challenging for her to seek help. The ongoing sexual encounters with the* pichaash *can leave her feeling powerless and vulnerable.***[Mata]**

Another traditional healer from Kailali district belonging to the Brahman/Chhetri ethnicity also believed that the problem with the character is possessed by a ghost called bayu, believed to be the spirit of someone who died unnaturally.

*She was afflicted by a wind* (bayu) *in her body. Bayu is a type of ghost that is believed to possess individuals who have died unnaturally, such as at a young age, by hanging, falling, burning, poisoning, being beaten, or dying suddenly.***[Dhami]**

Another traditional healer belonging to the Rajbanshi ethnic community in eastern Nepal, believed that the character in the vignette was suffering from the effects of a witch (*chudelni*), which was causing her symptoms. He explained that this issue is typically attributed to the spirit of a woman who died during childbirth. He emphasized the immense power of Chudelni*, describing them as capable of* halting a running motorcycle, transforming themselves into a beautiful girl, or killing a person in an isolated location.

*Laxmi [the character in the vignette] is being haunted by* chudelni*, a woman who died while trying to give birth. If a person is driving alone in a quiet location,* chudelni *can seize the motorcycle and throw it into a nearby river. Some* chudelni *may appear as charming girls and ask for a ride. If a person offers a lift to them, they may take the person to a riverbank and throw them into the river along with the motorcycle.***[Dhami]**

A Jyotish (astrologer) from Chitwan suggested that the problem faced by character in the story was caused by an evil eye. He explained that the evil eye can affect anyone regardless of age or species. The astrologer noted that the evil eye (*nazar*) can be recognized through observation, and there is a saying that goes ‘afflicted by the evil eye' (*nazar lageko*).

*Healthy children, animals, and birds becoming unhealthy because of an evil eye. Children can whine (*rohi rahane*), they may seem restless, and likewise, adult males or females and elderly people might have problems with indigestion, they may feel a burning sensation. Cattle like cows and buffaloes may stop giving milk. For all these types of problems, we try to protect them from such evil eyes.***[Jyotish]**

A clan traditional healer (*kul dhami*)) reported that the problem was caused by the clan god, *“kul devata,"* a deity worshipped by a specific caste or ethnic group for a long time. They believe that the clan god becomes angry when someone marries outside of their caste or ethnic group. During an interview the *kul dhami* demonstrated his supernatural powers when asked how he identifies a patient's disease, whether spiritual or physical. The interview had to be paused for approximately 10 min. Another traditional healer mentioned that the character's health issues may be attributed to Jangali, the goddess of the forest, believed to cause various health problems in those it possesses.

*It could be because of the goddess of the forest* (jangali)*. They are seven sisters, and these seven sisters can also cause such types of sufferings. It can make people become psychotic; they start to roll their eyes, their faces get paralyzed, become unconscious, and they start to feel that their bodies aren't working properly.***[Dhami]**

#### 3.1.2 *Husband's or ancestral spirit (*pitri dosh*)*

Some traditional healers reported that the issue in the character was attributed to a curse from her husband or ancestors, known as *pitri dosh,* which refers to the negative karmic debts of ancestors. They believe that if proper funeral rites are not performed for a deceased person or if they die in a location where rites cannot be conducted, the individual may not attain salvation, causing the spirit to cause trouble for the living. Similarly, they also reported that just as one can be affected by the karmic debts of a family member, a person can also suffer from their own past life sins.

*'*Pitri dosh *refers to a situation where a family member or guardian is unable to perform funeral rites. For instance, if funeral rites (e.g. funeral, 45-days rituals etc.,) are not conducted or if a person dies in a remote location, others may perform the rites but the deceased may not attain salvation or become an ancestor due to the lack of proper rituals. She is facing problems related to her blood. The source of her problem is her blood relatives who have been causing her stress.***[Dhami]**

Another traditional healer from Jhapa mentioned that the character in the vignette was experiencing such problems due to the unnatural death of her husband. They explained that when someone dies without fulfilling their desires, their soul may not rest in peace, causing trouble for the family members. The healer also mentioned that if the character, Laxmi, consults medical professionals, they may diagnose her problem as depression.

*Her husband may have died due to unfortunate circumstances, been killed in a painful manner, or passed away because of a series of unfortunate events. As a result, his spirit is restless and unable to find peace or salvation. This has put her in a stressful situation. She is already under a lot of stress, and the malevolent spirit (husband’s spirit) is also disturbing her. As a result, [the client] is feeling a lack of peace in her heart-mind, weakness, tingling and burning sensations in various parts of the body, rolling eyes, extreme lethargy, and a heavy, burdensome feeling akin to lifting a weight are all manifestations of spiritual influence.***[Bijuwa]**

#### 3.1.3 Personal and social factors

A few traditional healers suggested that the health issues of the character in the vignette were not caused by external factors like witchcraft or supernatural powers, nor were they due to a physical illness. They believed that the problem was linked to personal factors such as low self-esteem and confidence. A female traditional healer *(mata)* from Jhapa shared that her low self-esteem originated from losing her breadwinner, which made it challenging to care for her children in her husband's absence. While the healer acknowledged the character's struggles, she did not consider it an illness.

*She is not ill. She simply has low self-confidence, which led her to believe she was weak or ill. Being completely dependent on her husband, she was unprepared for his sudden death and the responsibilities that came with it. When one loses their own energy and self-belief, medical treatment from hospitals or doctors, as well as traditional healers, may not be effective in curing the patient.***[Mata]**

Similarly, a traditional healer from the Tharu community, an indigenous group in southern Nepal, reported that the character's issues could have multiple causes. The healer mentioned that the problems could be due to menstrual disorders, or heart-mind problems. However, he believed that heart-mind problems are personal issues and not disorders, so they do not require treatment as individuals with heart-mind problems do not face any issues.

*It is a menstrual disorder. Not feeling like eating and losing weight, skin getting darker and getting tired very quickly having no energy are the symptoms of menstrual disorder. If it is menstrual disorder, then going to the hospital won’t work.***[Guruwa]**

#### 3.1.4 Mental stress and thinking too much

Some traditional healers, particularly those with training in mental health suggested that the character's symptoms were related to mental health issues such as excessive thinking or mental stress. An astrologer from Chitwan stated that the character in the vignette was experiencing mental health issues due to stress and recommended engaging in enjoyable activities to help her forget past events that triggered her.

*She is dealing with mental health issues, specifically related to her thoughts and emotions. She often dwells on past events and prefers to be alone. When individuals are unable to find happiness in anything, they become sad, leading to mental health issues*. *Mental health problems can arise from various factors, including family pressure, challenging situations, and power of god (*bhagawan ko shakti*)*. **[Jyotish]**

A female traditional healer (*mata*), who has experienced depression in the past reported that the character's problems were related to mental stress caused by stress and lack of support. She noted that the character had not had the opportunity to share her inner feelings and emphasized the importance of seeking help and sharing problems.

*Based on your description, it seems likely that she is under significant mental stress or tension, possibly due to household responsibilities or everyday worries that are overwhelming her. Nowadays, it's even easier to say it is depression. People can experience depression without any apparent reason. I, too, became a traditional healer (*mata*) after experiencing depression. Therefore, even when you are under mental stress, you may quickly develop depression.***[Mata]**

A traditional healer (*kul dhami*) from Jhapa believed that the character's problems were caused by over thinking or mental pressure. He suggested that such issues often arise when a person is under pressure from someone else.

*This situation often occurs in individuals who tend to overthink, worry excessively, feel pressured, or are mentally distressed. They may isolate themselves and prefer to be alone. This isolation can lead to negative thinking and a lack of motivation to engage in activities. As their negative thoughts intensify, they may feel that everything is meaningless. Some individuals may even contemplate suicide as a way to escape their feelings of hopelessness.***[Kul dhami]**

Another traditional healer from the Tamang community also identified the character in the vignette as suffering from tension due to family burdens and lack of support. He explained that individuals experiencing constant tension and overthinking may struggle to fall asleep and lose their appetite. He emphasized that excessive worry and tension can lead to feelings of laziness and decreased appetite.

This is all due to tension, which means worrying too much. She doesn’t feel like working, possibly because of tension. She has to carry the burden of the family on her own and raise her kids after her husband's death. She also has to fulfill social responsibilities, which is difficult. She doesn’t have a breadwinner in the family, no one to rely on, and she has a lot of responsibility which may have caused tension. **[Lama]**

#### 3.1.5 Grief and stress due the death of husband

Some traditional healers suggested that the character's issues stemmed from the recent death of her husband causing her pain and distress. Lama, a traditional healer from the Tamang caste explained that stressful events, like losing loved one, can lead to excessive worry and overthinking, which can disrupt sleep. A 45-year-old male traditional healer from the Limbu community (*bijuwa*) believed that the symptoms of insomnia, fatigue, pessimism, gloominess, and feelings of worthlessness, arose after the character's husband pass way. He proposed that her illness may be connected to grief and stress from the loss of her husband.

*The story suggests that she is finding it difficult due to mental illness, maybe depression, as her husband had passed away, leading to economic difficulties and overthinking. The cause could be that after the death of her husband, she experienced pain* (pida) *from the loss, and if the pain accumulates, symptoms such as loneliness (*eklopan*), loss of appetite, and insomnia are triggered.***[Kul dhami]**

#### 3.1.6 Kundali (birth chart)

A 34-year-old male traditional healer linked Laxmi's health issues with her birth chart *(kundali),* and believed that life experiences are predetermined by it. According to him, any misfortune written in the *kundali* is inevitable and bound to happen. Laxmi's current difficulties were seen as a result of this predestined misfortune.

### 3.2 Assessment and treatment process

Traditional healers use various methods to assess and treat depression, guided by their belief systems and perceived causes of the issues. Assessment practices my involve examining the patient's face, using grains, invoking supernatural powers or spirits, reading birth charts, and tapping into psychic abilities to gain insight into the person's ailment. Traditional healers who believe a patient is experiencing mental stress or depression often recommend a combination of traditional healing and medical treatment for a faster recovery. They also stressed the significance of social support in boosting the patient's self-esteem and confidence.

#### 3.2.1 Using rice grains and praying

Many traditional healers rely on rice grains for assessing and treating patients. They count the grains on a plate and closely observe patients, starting from their eyes and then examining them from head to toe. This process helps them identify the causes of afflictions and find solutions. A traditional healer from Kailali reported that the cause of the problem in the character was *bayu (a type of ghost of a person who died unnaturally)* and that he treats patients using rice grains and prayers.

*While keeping that person in front and watching their eyes, the first thing to do is to call upon the gods in my own body. After calling upon all the known gods, I will experience a tremor for some time. Once the tremor subsides, I will take the rice grains they brought and divide them into two halves. These two rice grains will be used to determine if the person is afflicted or not. If the person is afflicted, they will touch one grain, and if not, they will touch the other grain without their knowledge. As soon as they touch the grains, I will say "okay, come." I will then throw the grains at them as an offering. If the person is afflicted, they may start speaking, claiming that they have been afflicted or describing their condition. They may also start trembling. I will then ask them questions such as "Who are you? What was not enough for you? Why did you trouble?" They must provide proof to support their claims. They must explain how they became afflicted.***[Dhami]**

The healers explain that their treatment methods are based on the spirit's response through the afflicted person. If there is no response from the spirit, they create a sculpture and transfer the illness to it.

*We ask what is lacking for the spirit, what it needs, etc. If it indicates that something is insufficient and requests a specific action, we comply. If there is no response, we create a mud sculpture, place fruits, and transfer the spirit to the sculpture. Once transferred, the person will be cured, and the issue will no longer affect them from the next day.***[Dhami]**

Another traditional healer from Jhapa reported similar practices to those of the traditional healer in Kailali. He mentioned that he receives divine power from God when he holds the rice brought by the afflicted person. He explained that he relies on his third eye to perceive beyond the material world. According to him, the third eye is essential for understanding what is happening in someone's inner being.

*I can identify the cause of suffering by focusing on grains of rice. Through prayer, I ask for god's guidance to open my senses. When I hold the grains of rice in the name of the afflicted person, I receive clarity and insight. I can determine if the suffering is due to their sins, witchcraft, evil spirits, or other sources. To perform this ritual, we use 21 rice grains divided into three groups. The first group is offered to gods and goddesses, the second to heavenly bodies, and the third to malevolent spirits. By observing with our third eye, we can identify if malevolent spirits or ghosts are involved.***[Bijuwa]**

#### 3.2.2 Using rice grain and communicating with gods

The female traditional healer from the Tamang community in Chitwan uses rice and incense sticks for diagnosis and treatment. She believes that the gods enter her body when she uses rice grains and burns incense sticks. Through this process, she communicates with the gods to identify the root cause of a person's suffering and prescribes the necessary materials to eliminate the illness from the person's body. Subsequently, she devises treatment plan based on this information.

*When I burn incense sticks and use rice grains, the gods will enter my body and communicate clearly. They will indicate their needs, where the items they need should be placed, where the spirit will go after leaving the body, and where it will stay. We converse with the spirit to determine the necessary items for its departure and make it promise to vacate the human body once all requirements are fulfilled and items are arranged as specified. After completing all tasks, we reexamine the person to confirm the spirit has departed.***[Mata]**

#### 3.2.3 Using smoke made from mustered, garlic and onion leaf

The traditional healer, who believed the character was affected by the evil eye, suggested that it could be removed by using a smoke made from mustard seeds, garlic, and dried onion leaves. He also recommended wearing a holy thread for protection against the evil eye in the future.

*The evil eyes* (nazar lagnu) *can be addressed by burning mustard seeds, garlic, and dried onion leaves to produce smoke. Holy thread* (baateko dhup) *helps to dispel negative thoughts. Black threads are tied around the necks of small babies, young children, and elderly individuals to shield them from ghosts and demons. Treatment is tailored to specific ailments.***[Jyotish]**

#### 3.2.4 Reviewing the birth chart

Some traditional healers believe that individuals' problems are influenced by the positions of the sun, moon, and other planets. They claim they can identify the planet affecting a person by reviewing their birth chart, which includes the date and time of birth. An astrologer from Jhapa explained that traditional healers have successfully treated various health issues by accurately interpreting astrological signs. When abnormal planetary positions are identified in a person's life, the healer recommends worshiping gods and engaging in religious activities. Worshiping deities in temples and burning incense in their honor are believed to alleviate many of these problems.

*I analyze individuals' birth charts, examining the positions of planets and their influences on various aspects of life. For example, the moon can impact mood, the Sun can affect health, and Mars can influence energy levels. By interpreting these planetary placements accurately, astrology can offer solutions to such issues. When planetary positions are unfavorable, I recommend religious practices such as worship and rituals to appease the Gods. Through temple visits and offerings like incense burning, many problems can be resolved in this manner.***[Jyotish]**

#### 3.2.5 Observing faces

A female traditional healer (*mata*) from the Tamang caste claims to diagnose illnesses by observing a person's face. She believes that through this observation, she can connect with the gods who will reveal everything about the patient.

*I can determine if a person is possessed by supernatural forces by observing their face. The patient sits in front of me, and I can discern their condition by simply looking at them. Once I make eye contact, I am able to connect with a god and receive insights into the person's situation.***[Mata]**

#### 3.2.6 Creating a clay statue

The traditional healer, who believed the character was experiencing a menstrual disorder, stated that he would provide various goods and services to appease the supernatural power causing her condition.

*We create a clay statue representing the person with a menstrual disorder and instruct them to bring makeup tools such as a comb, mirror, and various cosmetics. The patient does not need to come to us; we perform the ritual ourselves. We sculpt the clay figure, add hands and legs, apply vermilion on the forehead, kohl on the eyes, and place dirt, fingernails, and rice as offerings. A few drops of alcohol are also included. We inform the spirit that we have fulfilled their requests and urge them to stop causing harm, warning of potential consequences if they continue. As humans, we consider ourselves inferior to the spirits and strive to carry out their wishes to appease them.***[Guruwa]**

#### 3.2.7 Listening to patients’ sufferings

Some traditional healers assess and treat patients by listening to their suffering and symptoms, providing support as needed. They emphasized the importance of boosting self-confidence *(aatma bishwas)* and receiving support from others to recover from suffering. A female traditional healer *(mata)* who identified mental stress as the issue recommended social support as the most effective treatment method for relief.

*Some find relief from mental stress through worship, while others find peace in solitude. Receiving social support is crucial for fulfilling one's wishes. If she receives social support then her thoughtful wishes will be fulfilled. Without social support, effective treatment may not be possible.***[Mata]**

#### 3.2.8 Getting rid of pitri-dosh

Many traditional healers use various methods to remove ancestral curses, each with its own specific procedure. For instance, if an illness is believed to be caused by negative karmic debts of ancestors, a ritual called pitri-dosh is performed. Traditional healers attribute the issue to ancestral spirits and recommend a ritual involving specific items to bring salvation to the deceased ancestors. They assure that the afflicted person will fully recover once the ancestral curse is removed.

*To perform the ritual for* 'pitri-dosh'*, we gather a lotus flower, create* 'sapta-rishi' *idols, and prepare 365* 'pinda' *offerings using barley flour. Proceed to a river and construct a* 'trikuti' *structure using plants like* "paiyu" *or* "simali". *Fill the* 'pinda' *with milk through the* 'trikuti' *and recite* ‘namanarayan’ *before releasing them into the river. This act will grant salvation to the deceased ancestors, allowing them to transition to their realm through the river. The individual performing the ritual will witness a full recovery to their previous state.***[Dhami]**

Another traditional healer, who believed that the character's illness was caused by the husband's spirit, reported that the husband's soul had not found salvation. He recommended a Hindu ritual called “Purna-path" which worships the god Vishnu as a symbol of truth and righteousness. Purna-path is a performed to remove the negative effects of misfortunes and help the deceased attain salvation. Common types of purna-path suggested by traditional healers include ‘Satyanarayan puja' or ‘Narayanbali puja'

*The husband has passed away, and while we cannot bring him back, we must ensure the well-being of the wife and children. To achieve this, a 'Purna-Path' must be conducted in their home. For instance, if the spirit has not found salvation, we can perform a* 'satyanarayan puja' *or* 'narayanbali Puja'*. This ritual can be carried out with either 8 or 16 priests. With 16 priests, the focus is on Lord Shiva, while with 8 priests, it is on 'Aadhi Anga', which represents the Aghori tradition. By using 16 priests and invoking Lord Shiva during the* 'Narayanbali Puja'*, the family members can find salvation and potentially alleviate misfortunes. This is one approach to address the situation.***[Bijuwa]**

#### 3.2.9 Shouting (jharfuk)

Another method of treatment involves shouting at the evil spirit to leave the body of the sick person. Some traditional healers in Nepal use this approach, known as *jharfuk*, for treatment.

*I derive my strength from a goddess, allowing me to handle any situation quickly. I do not need to use sacred grains* (akshatata). *By simply commanding, "leave the patient," the illness vanishes*. **[Kul dhami]**

#### 3.2.10 Chanting mantras

Some traditional healers use sacred words *(mantras)* to treat patients. They assess patients based on the information shared and then use spells and exorcism for healing. One traditional healer mentioned that medical treatment may not be effective if a spirit is present in the body. Only after expelling the evil spirit through exorcism or spells can medical treatment be effective.

*We need to perform exorcisms accurately to heal the illness. By removing the negative energy of the spirit, the patient will be freed from its influence. Once the spirit's energy is removed, medical treatment will be more effective as the patient will no longer be affected by the spirit's energy.***[Bijuwa]**

#### 3.2.11 Using a pair of pigeons

A traditional healer, who believed the character was possessed by a chudelni, a spirit of a woman who died unable to give birth, required a pair of pigeons for the treatment. He explained that the chudelni would kill the pigeons with her ten hands during his blowing ritual. He advised that the process should be repeated for three days to fully remove the bad spirit.

*I need a pair of pigeons to help treat a person possessed by* chudelni*. The pigeons absorb the sickness and unfortunately die in my hands.* Chudelni*, with ten hands, kill the pigeons. I place cotton wicks in the west direction, make offerings in nine different places, and have the patient pour water on them. I then perform repetitive blowing, and after three blows, the patient makes a sound "Haa" to indicate the ghost has left the body. This process must be repeated daily for three days for the patient to fully recover.***[Dhami]**

The same traditional healer explained that if the spirit *(chudelni)* enters an individual's brain, traditional healing alone may not be effective. In such case, combination of medical treatment and traditional healing is necessary.

*If ghosts* (chudelni) *enter the brain, the person may become mentally ill. If the brain is weakened, medical treatment is essential. It is important to take the person to a doctor who can assess the brain's condition and prescribe appropriate medication. The medication may cause drowsiness, but it is necessary for the person's recovery. In addition to medical treatment, traditional healing methods such as Jharfuk and consulting a traditional healer (dhami) may also be recommended. The patient should continue taking the prescribed medication and seek traditional treatment simultaneously to facilitate recovery.***[Dhami]**

#### 3.2.12 Referring to a doctor/psychologist

Some traditional healers, especially those who believe that the issues are related to mental health problems, reported that they refer such cases to doctors or psychologists. They believe that the problem cannot be treated by traditional healing methods alone. They also emphasized the importance of improving the home environment, as unresolved sources of stress at home may hinder the effectiveness of medical treatment.

*I recommend advising them, "You should not visit a traditional healer* (dhami jhankri)*; instead, see a doctor." The first recommendation is to participate in refreshing and engaging activities. The second suggestion is to seek medical advice. By following these steps, their symptoms will improve. The third recommendation is to create a supportive environment at home. Even after consulting with a doctor, the patient may not recover if there is stress and tension in the household.***[Kul dhami]**

The traditional healer, who felt that the character had mental health issues, recommended seeking help from psychologist or a hospital.

*I have previously mentioned that this individual has a mental illness. Therefore, she should seek treatment from a psychologist. Even if such patients consult me, I recommend that they seek treatment at a hospital.***[Kul dhami]**

Another traditional healer, who identified mental stress as the issue, recommended that patients consult a doctor before seeking traditional healing. He believed that a combination of medical treatment and traditional healing practices could lead to better recovery.

*My first advice is to prioritize visiting a doctor before seeking help from a traditional healer like me. I recommend this approach because I am also a traditional healer and believe in the importance of medical treatment. If the illness persists after seeing a doctor, then she can come to me for further assistance. This has been my practice so far.***[Mata]**

#### 3.2.13 Offering support (counseling)

The female traditional healer *(mata)* explained that there is no specific treatment for the character's condition, but addressing the root causes of her problems is crucial. She recommended providing support, encouragement, and practical advice to improve her life. Mata also suggested that counseling to boost the character's self-confidence and help her adjust to life without her husband would be more beneficial than medical treatment. She emphasized the importance of family support in the character's recovery.

*Until we can boost her self-confidence, we won't be able to help her overcome her illness. She is not actually ill; she simply lacks self-confidence. Her low self-esteem and sense of powerlessness make her feel weak and unwell. If she had family members who could provide counseling, she might not be facing this issue.***[Mata]**

## 4 Discussion

This study explored the beliefs and treatment practices related to depression among traditional healers from different castes and ethnic groups in Nepal. Individual interviews were conducted with 12 traditional healers from Jhapa, Chitwan, and Kailali districts using a narrative approach. The findings revealed diverse perspectives on the causes and treatment of depression. Some participants attributed depression to supernatural forces such as ghosts, spirits, or jungle gods, while others viewed it as a result of mental stress or life events. Some traditional healers linked depression to the sorrow and trauma experienced by a character due to the sudden death of her husband, while others associated it with Pitri-Dosh or unrestful spirits of the deceased husband. Variations were also observed in the assessment and treatment approaches used by different traditional healers. Traditional healers with higher education or those who identified lack of social support, personal/family circumstances, and traumatic experiences as causes of depression recommended treatments like emotional support, counseling, or referral to medical professionals. Some traditional healers suggested traditional treatments involving offerings to gods, while others emphasized the importance of performing rituals correctly or removing negative energy through various methods.

The study has some important findings. Firstly, many traditional healers correctly identified the vignette as depicting depression or mental stress, rather than attributing it to supernatural causes. They believed that the character in the vignette was experiencing depression, mental stress, or tension. They linked the problems to stressors in daily life, overthinking, increased household responsibilities due to the husband's death, and lack of support from family and the community. The healers acknowledged the importance of seeking medical help for such conditions and would refer cases to hospitals or mental health specialists such as psychiatrists or psychologists when their problems did not improve with their treatment. They also emphasized the need for a combination of medical treatment and traditional healing practices for full recovery. These findings align with previous studies showing that patients often seek medical care after consulting with traditional healers (Pandey, 2024).

Another key finding of the study is that an equal number of traditional healers believed that the problem was caused by supernatural powers, the spirit of a deceased person, or a clan god, which is consistent with a previous study conducted in Western Nepal (Khanal et al., 2020). Despite differences in the perceived causes of depression, there was consensus among traditional healers regarding the impact and symptoms of the problem. All traditional healers agreed that individuals experiencing such situations may have disturbances in sleeping, difficulties in concentration, changes in appetite, psychosomatic symptoms like headaches, stomachaches, loneliness, and thoughts of death or suicide. The symptoms reported by traditional healers are also consistent with the common symptoms of depression reported by patients receiving treatment from primary care (Luitel et al., 2024), and are often expressed through physical symptoms such as headaches and stomachaches (Luitel et al., 2017). Previous studies have indicated that individuals with depression or anxiety often seek help from traditional healers before consulting medical professionals (Pandey, 2024; Thapa et al., 2018). This may explain why most traditional healers treat individuals with these issues on a daily basis.

Traditional healers have been known to administer harmful and costly treatments, such as extreme physical reactions, vein popping, threats, waterboarding, physical assault, and applying red-hot irons to patients, resulting in serious injuries (Jimba et al., 2005; van Leeuwen, 2008). However, the treatments recommended by the healers in this study were not harmful or costly (Pham et al., 2021a). Most of the treatments suggested by traditional healers are psychological, focusing on building self-esteem or promoting long-standing rituals that are acceptable. Traditional healers who attribute problems to supernatural forces like *Mashaan, Pichaash*, ancestral curses, or evil spirits also believe that doctors would diagnose such conditions as depression if consulted. This indicates that these healers may have knowledge about depression and provide treatments that are not physically or psychologically harmful.

The findings of this study have implications for improving help-seeking behavior for mental health care in Nepal. First, traditional healers who have received brief training in mental health reported referring individuals with mental health problems to hospitals or specialists and emphasized the importance of family support to build self-confidence and self-esteem. They identified the character in the vignette as experiencing depression, mental stress, or tension. Collaborating with traditional healers through training can help identify individuals with severe mental health issues and imminent risk of suicide, and refer them to trained primary health care workers or mental health specialists. Traditional healers can continue providing services to those with milder forms of illness by themselves, as previous literature has also shown that traditional healing has therapeutic values (Pham et al., 2021a). Research shows that people trust traditional healers and are more likely to seek help from healthcare providers recommended by them (Thapa et al., 2018). Second, traditional healers who attribute possession to supernatural or divine causes for mental health issues often describe symptoms similar to depression. Training them to refer individuals with these symptoms to trained primary healthcare workers or mental health professionals can improve access to services. Traditional healers also acknowledge that their treatments may not always lead to full recovery. Previous studies have reported that individuals experiencing depression, anxiety, or stress tend to seek help from traditional healers before visiting medical professionals since the treatments provided by them are affordable and culturally relevant within the community (Pandey, 2024). Establishing a two-way referral system between traditional healers and medical professionals can enhance the recovery process, build trust in available services, and boost the confidence of traditional healers in their practice. Third, most of the treatment options recommended by traditional healers, who believe the issue stems from supernatural powers, appear culturally relevant and non-harmful. Traditional healers associate the character's symptoms with supernatural or divine power or curses from the husband or ancestors' spirits. They suggest cultural rituals such as worshiping gods, removing bad spirits from the body, or performing death rituals properly to address these issues. These treatment options align well with existing customs that have been practiced for a long time. Previous research has also shown that widows often experience sorrow and stress until these rituals are completed within a year (Kim et al., 2023). Therefore, promoting the treatments recommended by traditional healers could help continue these long-standing practices.

The study has several limitations. Firstly, it involved twelve traditional healers from Jhapa, Chitwan, and Kailali districts, representing the eastern, central, and far western regions of Nepal. Nepal is a diverse country with over 125 caste/ethnic groups, each with its own cultural and traditional healing practices. These districts were selected due to their diverse and populous communities. It is important to acknowledge that traditional healing practices in hilly and mountainous districts may differ from those in the study sites. Secondly, the study was conducted as part of a research grant aimed at developing and optimizing an intervention to promote help-seeking for mental health care. Therefore, other aspects of traditional healers, such as their willingness to receive mental health training, collaborate with healthcare providers, and their perspectives on why people with mental health problems do not seek treatment from medical professionals, were not explored. Thirdly, the interviews were guided by the story of a female character whose husband recently passed away, and is facing challenges in taking care of her children and managing household responsibilities. Participants' perceptions may have been influenced by the gender, age, and other socio-economic aspects of the character presented in the vignette. Finally, due to a lack of patient interviews, the information provided by traditional healers could not be verified. Future studies should involve both traditional healers and their patients to verify the assessment and treatment provided by them.

The results indicate that traditional healers who attribute depression to supernatural forces tend to prefer traditional treatment methods. However, a combination of medical and traditional healing approaches is often preferred, as some believe that medical treatment alone may not be effective without addressing negative energy in the body. Collaborating with traditional healers, who act as gateways to mental health care, could enhance early detection and treatment of depression. Certain traditional healing practices may provide benefits for depression, so involving them in identifying and supporting individuals with depression could be beneficial. Future research should evaluate the effectiveness of traditional healing treatments and explore patients' perspectives on these treatments.

## CRediT authorship contribution statement

**Nagendra P. Luitel:** Writing – review & editing, Writing – original draft, Supervision, Project administration, Methodology, Investigation, Funding acquisition, Formal analysis, Conceptualization. **Bishnu Lamichhane:** Writing – review & editing, Validation, Methodology, Data curation. **Prakriti Koirala:** Writing – review & editing, Validation. **Poonam Sainju:** Writing – review & editing, Formal analysis, Data curation. **Amshu Ghimire:** Writing – review & editing. **Kamal Gautam:** Writing – review & editing. **Mark JD. Jordans:** Writing – review & editing, Methodology, Investigation, Conceptualization. **Brandon A. Kohrt:** Writing – review & editing, Methodology, Investigation, Conceptualization.

## Ethical standards

The study has received ethical approval from the Nepal Health Research Council (NHRC) (Registration number: 527/2022 P).

## Availability of data and materials

Interested individuals can contact the principal investigator of this study to express their interest in collaboration and request access to the dataset analyzed here by emailing: luitelnp@gmail.com.

## Financial support

This work was supported by the National Institute for Health Research (NIHR) (using the UK's Official Development Assistance (ODA) Funding) and Wellcome [222001_Z_20_Z] under the NIHR-Wellcome Partnership for Global Health Research. The views expressed are those of the authors and not necessarily those of Wellcome, the NIHR or the Department of Health and Social Care.

### Declaration of generative AI and AI assisted technologies in the writing process

During the preparation of this work, the author (s) did not use AI or AI assisted technologies in the writing process.

## Declaration of competing interest

The authors declare that they have no known competing financial interests or personal relationships that could have appeared to influence the work reported in this paper.
